# Network analysis reveals that the tumor suppressor lncRNA GAS5 acts as a double-edged sword in response to DNA damage in gastric cancer

**DOI:** 10.1038/s41598-022-21492-x

**Published:** 2022-10-31

**Authors:** Shantanu Gupta, Pritam Kumar Panda, Wei Luo, Ronaldo F. Hashimoto, Rajeev Ahuja

**Affiliations:** 1 Departamento de Ciência da Computação, Instituto de Matemática e Estatística, Universidade de São Paulo, Rua do Matão 1010, São Paulo, SP 05508-090 Brasil; 2 Condensed Matter Theory Group, Materials Theory Division, Department of Physics and Astronomy, Uppsala University, Box 516, 751 20 Uppsala, Sweden; 3 Department of Physics, Indian Institute of Technology Ropar, Rupnagar, Punjab 140001 India

**Keywords:** Bioinformatics, Biological models, Computer modelling, Dynamic networks, Regulatory networks, Computational biology and bioinformatics, Systems biology

## Abstract

The lncRNA GAS5 acts as a tumor suppressor and is downregulated in gastric cancer (GC). In contrast, E2F1, an important transcription factor and tumor promoter, directly inhibits miR-34c expression in GC cell lines. Furthermore, in the corresponding GC cell lines, lncRNA GAS5 directly targets E2F1. However, lncRNA GAS5 and miR-34c remain to be studied in conjunction with GC. Here, we present a dynamic Boolean network to classify gene regulation between these two non-coding RNAs (ncRNAs) in GC. This is the first study to show that lncRNA GAS5 can positively regulate miR-34c in GC through a previously unknown molecular pathway coupling lncRNA/miRNA. We compared our network to several *in-vivo/in-vitro* experiments and obtained an excellent agreement. We revealed that lncRNA GAS5 regulates miR-34c by targeting E2F1. Additionally, we found that lncRNA GAS5, independently of p53, inhibits GC proliferation through the ATM/p38 MAPK signaling pathway. Accordingly, our results support that E2F1 is an engaging target of drug development in tumor growth and aggressive proliferation of GC, and favorable results can be achieved through tumor suppressor lncRNA GAS5/miR-34c axis in GC. Thus, our findings unlock a new avenue for GC treatment in response to DNA damage by these ncRNAs.

## Introduction

Gastric cancer (GC) is responsible for the second-highest number of cancer-related deaths worldwide, making it a major medical issue and an attractive target for drug development^1^. Dysregulation of dominant signaling pathways through alterations in key oncogenes and tumor suppressors is one of the hallmarks of GC, which initiates aberrant cell proliferation and survival^2^. In this context, Zheng et al.^3^ provide evidence that E2F transcription factor 1 (E2F1) remains highly expressed in GC and is accountable for GC tumorigenesis in BGC-823, MGC-803, and SGC-7901 cell lines. Furthermore, Zheng et al.^3^ found that microRNA-34c-5p (hereinafter referred to as miR-34c) is downregulated in the same GC cell lines. Interestingly, Zheng et al.^3^ determined that E2F1 directly inhibits miR-34c expression at the transcriptional level. Furthermore, Zheng et al.^3^ confirmed that knockdown (KO) of E2F1 triggers miR-34c expression, whereas overexpression (E1) of E2F1 shows the opposite result. In conclusion, Zheng et al.^3^ showed that upregulated miR-34c could suppress GC proliferation through induction of cell cycle arrest and apoptosis at the G1/S checkpoint following E2F1 knockdown.

The miR-34 family (miR-34a, miR-34b, and miR-34c) is a master regulator of tumor suppression and plays an essential role in the DNA damage response^4^. MiR-34c is downregulated in several types of cancer, including GC^3^. The transactivation of miR-34c by p53 is well known, although new evidence has shown that miR-34c activation occurs through the ATM^4^ or ATM/p38 MAPK pathway^5^, which is independent of *de-novo* p53-mediated transcription^4,5^. Furthermore, the study of Suzuki et al.^6^ provides evidence that miR-34b and miR-34c are found to be epigenetically silenced in GC cell lines. Moreover, Suzuki et al.^6^ further reported that there is no correlation found between miR-34b/c and p53 functionality in GC. Interestingly, it is well-recognized that cyclin-dependent kinase inhibitor 1 (p21) is transactivated through p53. Nevertheless, in GC cell lines, p21 was found to be governed by ATM/p38 pathway. In more detail, Liu et al.^7^ found that activation of p21 was strongly detected in GC cell lines with Wildtype (WT) p53 (MKN-45) and cells lacking WT p53 (MKN-28). Moreover, Liu et al.^7^ further explained that p21 was found to act as a switch between cell cycle arrest and apoptosis due to its ability to inhibit caspase 3 activity in GC cell lines. Furthermore, upregulation of p21 was determined through the p38 MAPK signaling pathway rather than p53^7^. On the other hand, Subhash et al.^8^ provide evidence that p53 is not essential for cell cycle arrest and apoptosis in the G1/S checkpoint in GC. In more detail, Subhash et al.^8^ observed that ATM is required to induce cell fate decisions such as cell cycle arrest and apoptosis in GC cell lines (IM95, IST1, NUGC4, TMK1, YCC3, MKN45, and MKN1) at the G1/S checkpoint. Moreover, Subhash et al.^8^ found that overexpression of ATM in response to DNA damage induces robust induction of p21 and PUMA in CG cell lines, independently of p53. Interestingly, miR-34c regulates p21 expression independent of p53 in various cancer cell lines^9^.

It is well known that protein-coding genes account for above than 1% of the total genome, whereas large portions of the human genome can be transcribed into non-coding RNAs (ncRNAs)^10,11^. Indeed, miRNAs may be sufficient to inhibit proliferation in GC cells in response to DNA damage^12^. miRNAs are a class of small ncRNAs often associated with several biological processes such as cancer progression^12^. Recent studies identified a novel class of small ncRNAs, i.e., long noncoding RNAs (lncRNAs), which may regulate cell proliferation in GC through downstream DNA damage mechanisms^13^. lncRNAs have recently emerged as important players in cancer biology and can be used for cancer diagnosis, prognosis, and potential therapeutic targets^13^. In this context, a tumor suppressor lncRNA-GAS5 (growth arrest-specific transcript) was found to be dysregulated in various cancers, including GC^14^. Furthermore, in response to DNA damage, overexpression of the lncRNA-GAS5 can suppress cancer progression by induction of cell-cycle arrest or cellular apoptosis in many types of cancer, including GC^14^. Interestingly, recently, Sun et al.^15^ exposed the role of lncRNA-GAS5 in (SGC7901, BGC823, MGC803, MKN45, MKN28) GC cell lines. In detail, Sun et al.^15^ found that lncRNA-GAS5 expression was markedly downregulated in GC tissues and correlated with larger tumor size. Moreover, these authors^15^ further demonstrated through the *Vivo/Vitro* experiments that gain-of-function (GoF) of lncRNA-GAS5 inhibits gastric cancer cell proliferation by inducing cell fates at the G1/S checkpoint, while downregulation of lncRNA-GAS5 could promote cell proliferation. Furthermore, Sun et al.^15^ confirmed that E2F1 and G1/S-specific cyclin-D1 (Cyclin D1) were functional targets of lncRNA-GAS5 in GC cells. However, the advanced aspect of the lncRNA-GAS5 in the coordination of miR-34c expression in response to DNA damage at the G1/S checkpoint remains unexplained in GC cells.

Boolean models of regulatory networks were meant to simplify the dynamics of complex biological systems^16^. This method provides a qualitative description that captures advanced features of network dynamics^17^. For example, enclosed pathways connecting two or more nodes in a network (similar to feedback loops in the continuous model) may serve as regulatory circuits controlling the dynamics of the network^18–21^. Classification of molecular regulatory networks in a rational framework by a computational-experimental combinatorial process is recognized as a valuable approach for cell fate choice and the study of various biological processes^19,20,22–28^. For more details about Boolean modeling, see the Methods section.

**Figure 1:**
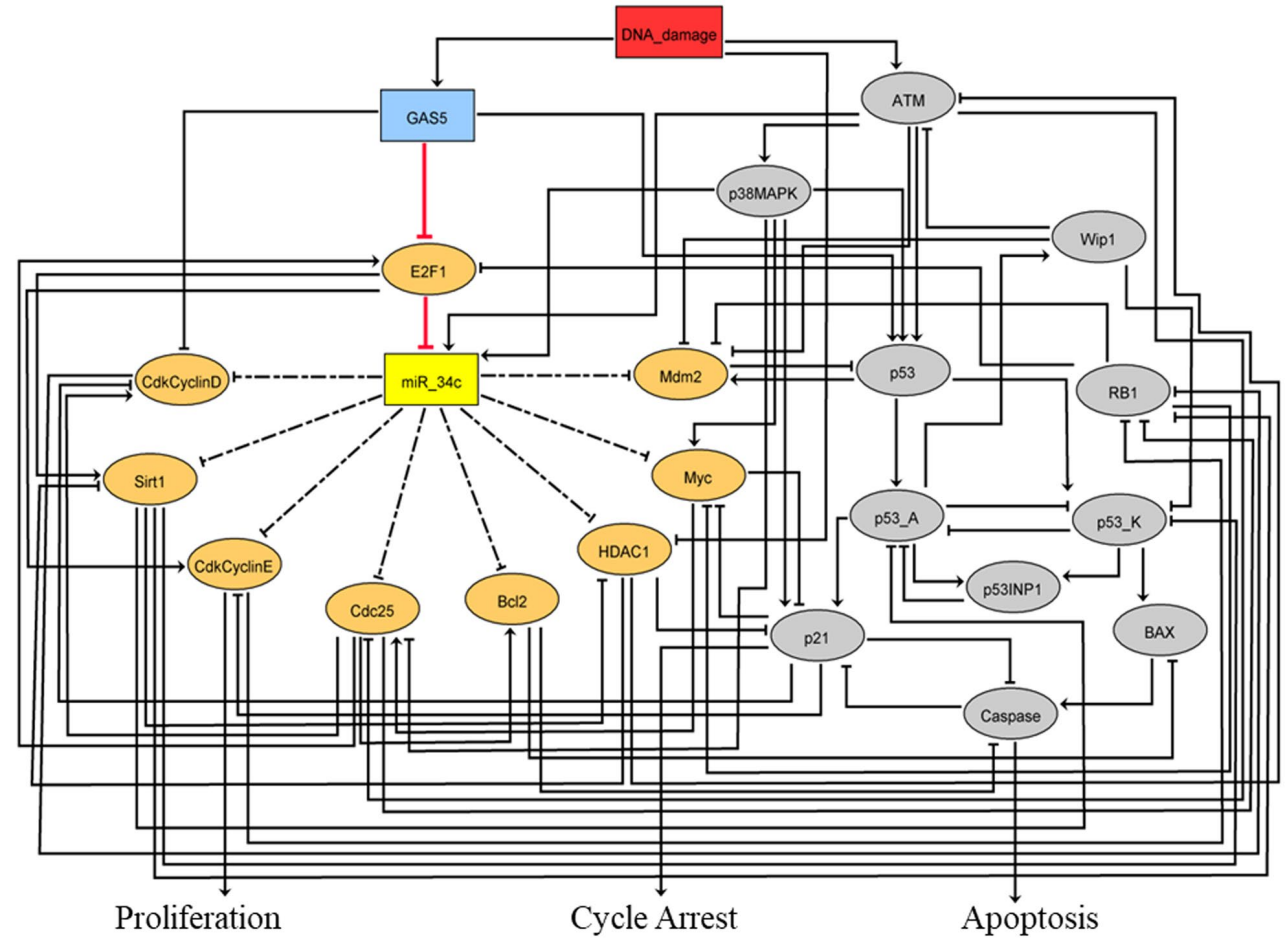
Gene regulatory network of lncRNA-GAS5 for the GC. Arrows indicate activations, and hammer-head arcs indicate inhibitions, respectively. The blue rectangular node represents lncRNA-GAS5, the yellow rectangular node represents miR-34c, and the red one represents DNA damage which is the input to the Boolean model. Dashed hammer-head arcs represent the targets of miR-34c highlighted by orange-colored oval nodes while other proteins are in grey-colored nodes. Model outputs are proliferation (in the lack of DNA damage) or cell cycle arrest, or apoptosis in the occupation of DNA damage.

Inspired by the facts mentioned above^3,15^, in this work, we proposed a mathematical model to uncover the unique function of the lncRNA-GAS5 in the gene regulation of GC at the G1/S checkpoint (see Fig. 1). Indeed, to our knowledge, this is the first fundamental study that uncovers a positive concrete nexus between lncRNA versus miRNAs, i.e., lncRNA-GAS5 controls miR-34c expression in GC.

## Results

### Boolean model and its fixed points

There are 26 nodes in our Boolean network. The blue rectangular node represents lncRNA-GAS5, the yellow node signifies miR-34c, and the red one defines DNA damage which is the input of the Boolean network. Dashed hammer-head arches are highlighted by orange-colored oval nodes delineating the targets of miR-34c, while other proteins are in grey-colored nodes. The three outputs (in white nodes) of the network are proliferation, cell cycle arrest, and apoptosis, respectively. There are a total of 73 direct interactions within these nodes. See Fig. 1.

**Figure 2:**
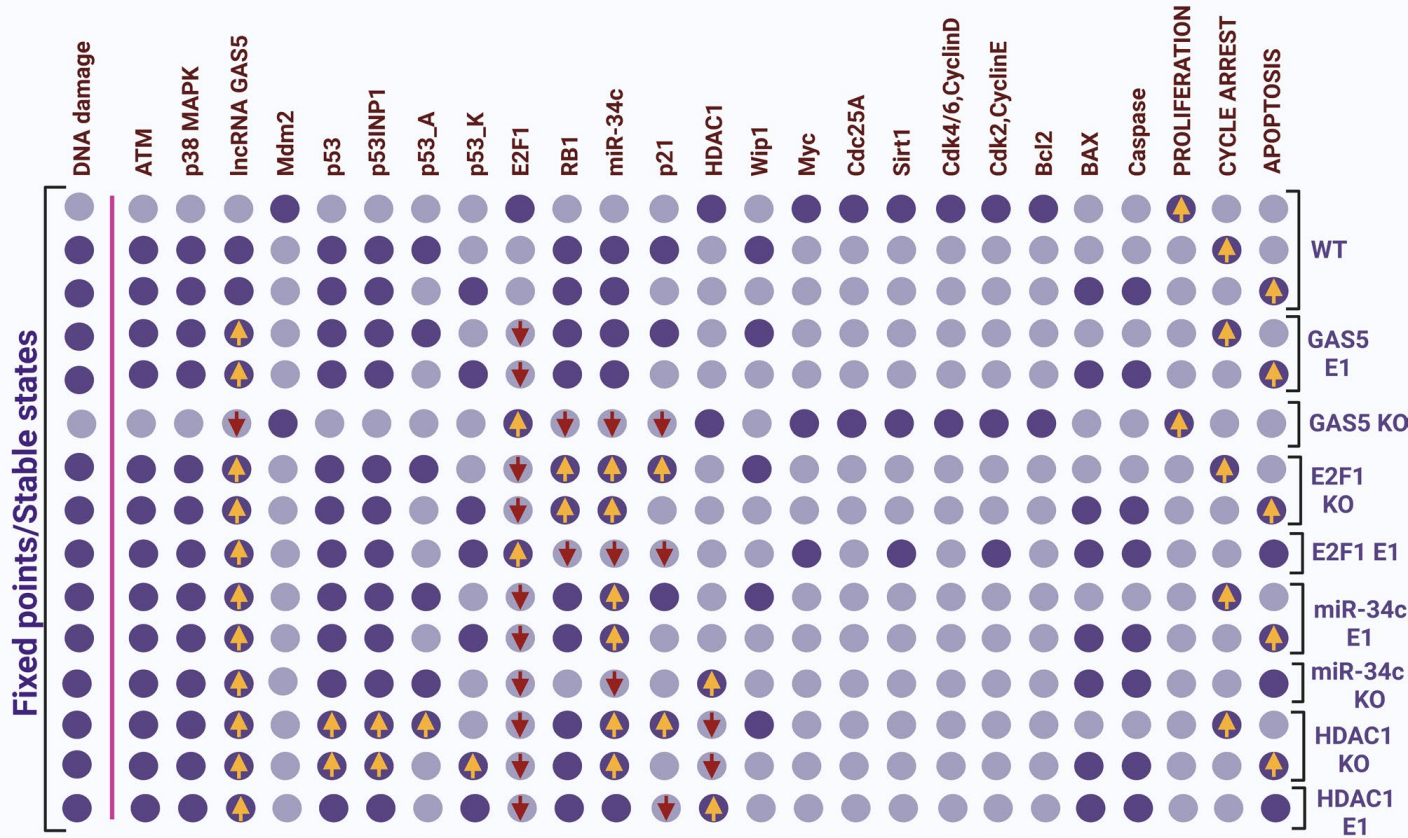
Model fixed points for WT case and perturbations. The fixed points identified for distinct scenarios: WT, GAS5 E1, GAS5 KO, E2F1 KO, E2F1 E1, miR-34c E1, miR-34c KO, HDAC1 KO, HDAC1 E1, and p53 KO. Ectopic expression (E1) represents gain-of-function (GoF) while knockdown (KO) represents loss-of-function (LoF) of the respective network element. The leftmost column shows the level of DNA damage with other molecules separated by a pink line. At the same time, the rightmost columns show the model outputs: proliferation, cell cycle arrest, and apoptosis. Each line represents a fixed point or steady state corresponding to the input. Light purple cells indicate a zero value (inactivation), while dark purple cells indicate activation (value 1). The first three fixed points belong to the wild-type case. The remaining fixed points explain how GAS5 regulates the miR-34c expression, and at the end, three fixed points linked to the HDAC1/p21 pathway represent activation of p21 via knockdown of HDAC1 by miR-34c. The GoF of GAS5 accelerates miR-34c expression that triggers cell cycle arrest and apoptosis, whereas knockdown (KO) of GAS5 inhibits miR-34c expression but induces E2F1 that, triggers proliferation. Knockdown (KO) of E2F1 increases miR-34c expression that induces cycle arrest and apoptosis, whereas its gain of function (GoF) inhibits miR-34c expression. Overexpression of  miR-34c induces cell cycle arrest and apoptosis, whereas knockdown (KO) triggers apoptosis. Next, the knockdown (KO) of HDAC1 enhanced p21 expression, which means induction of cell cycle arrest, and apoptosis. In comparison, its gain-of-function (GoF) inhibits p21 expression and induces apoptosis. Yellow arrows represent upregulation, while the red arrows facing down represent downregulation, respectively.

Our network simulations show 3 fixed points for the wild-type case dynamics (WT) that are associated with multiple phenotypes, Fig. 2. The first fixed point indicates a proliferative state (corresponding to input: DNA damage = OFF), i.e., no cycle arrest is identified, only cell cycle enhancers are active: cdk4/6-CycD, cdk2/CycE, Cdc25A, HDAC1, Myc and E2F1. Additional two fixed points (produced by the same input: DNA damage = ON), i.e., cell cycle arrest and apoptosis, respectively. Interestingly, all these three fixed points are consistent with in-vivo/in-vitro experiments^3,15^ in GC.

### lncRNA-GAS5 modulates miR-34c expression through the targeting of E2F1

To uncover the advanced aspect of lncRNA-GAS5 in the regulation of miR-34c expression through the knockdown of E2F1, which is the main focus here. We interrogated lncRNA-GAS5/E2F1 and miR-34c, i.e., we performed GoF/LoF perturbation. The results are presented in Fig. 2. We found that overexpression (E1) of lncRNA GAS5 diminished E2F1 expression and increased the expressions of miR-34c, RB1, and p21. In comparison, knockdown (KO) of lncRNA GAS5 gives the opposite effect. On the other hand, overexpression of E2F1 declined miR-34c, RB1, and p21 expressions. At the same time, the knockdown of E2F1 presents opposite outcomes. In the last, overexpression (E1) of miR-34c inhibits proliferation through the induction of cell cycle arrest and apoptosis. Whereas knockdown (KO) of miR-34c enhanced apoptosis. As can be seen in Fig. 2, overexpression (E1) or knockdown (KO) of miR-34c did not affect the lncRNA-GAS5 expression.

Interestingly, all these results are in excellent agreement with in-vivo/in-vitro experiments^3,15^ in GC. Our results suggest that lncRNA-GAS5 is an important regulator of miR-34c activity in GC. Additionally, these results further pinpointed that lncRNA-GAS5 can also regulate RB1 and p21 expressions in GC.

### Induction of p21 through the knockdown of HDAC1 by miR-34c

HDAC1 is a well-known tumor promoter in GC cells. Growing evidence suggests that targeting HDAC1 can upregulate p21 by miR-34c^9,29,30^. Therefore, we conducted the node perturbations to examine the similarity between phenotypes (model attractors) and experimental observations. Results are presented in Fig. 2. We found that the direct suppression of HDAC1 due to overexpression of miR-34c induces p21 expression, while the gain-of-function (GoF) of HDAC1 downregulates p21 (see Fig. 2). Additionally, Liu et al.^7^ provide evidence that p21 acts as a switch between cell cycle arrest and apoptosis, and the functionality of this switch does not depend on p53 status, implying that p21 can regulate cell cycle arrest and apoptosis in p53-deficient GC cell lines. To test it, we carried out another perturbation of loss-of-function (LoF) of p53. Results are presented in Fig. 2. These results show that miR-34c indirectly regulates p21 in GC. Thus, miR-34c functions to regulate cycle arrest and apoptosis through the HDAC1/p21 pathway that does not depend on the p53 functionality. Our results are in the finest agreement with these in-vivo/in-vitro experiments^7,9,29,30^.

### The ATM/p38 MAPK pathway is required for the lncRNA-GAS5-mediate cell fate decision in GC

**Figure 3:**
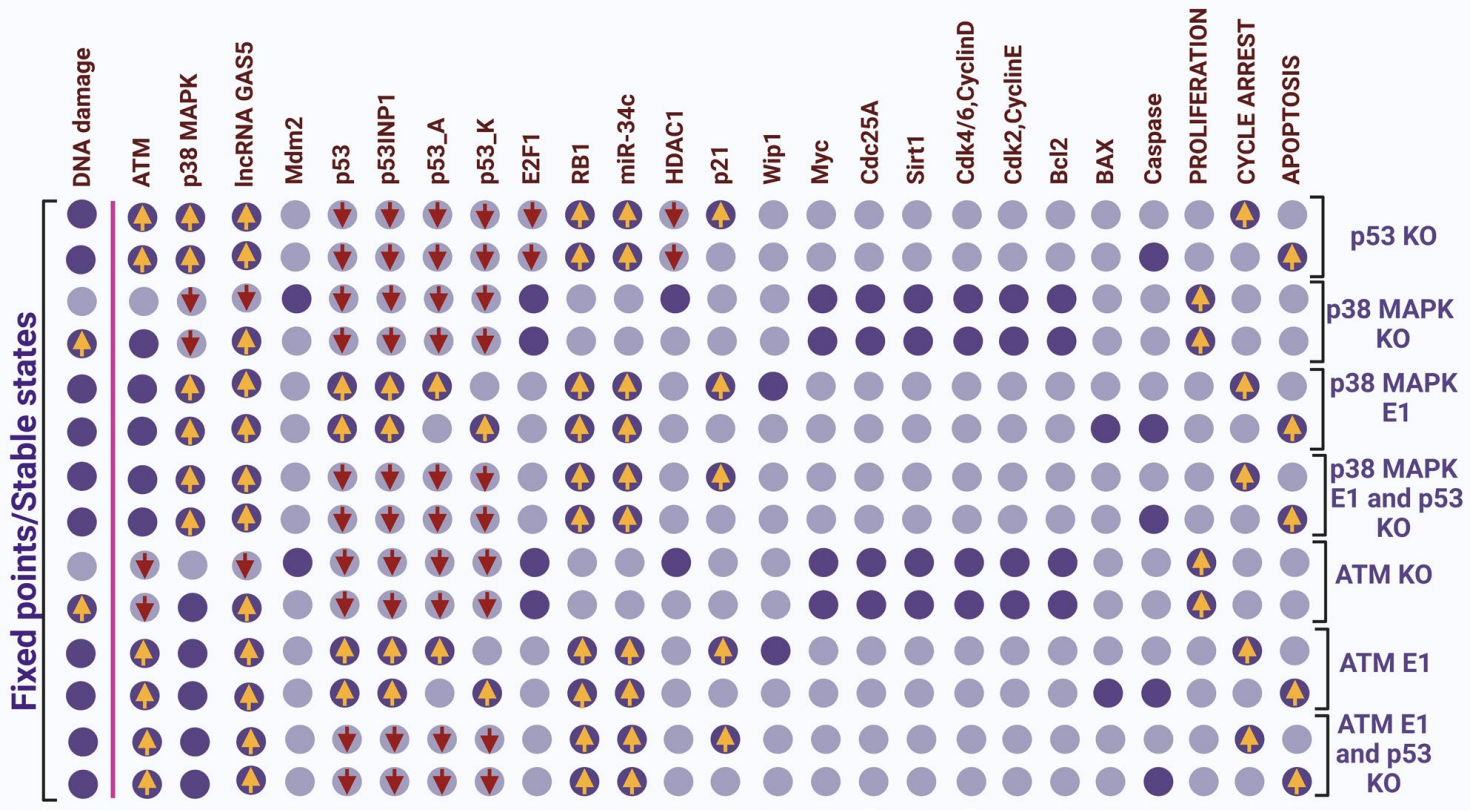
Interrogation of the ATM/p38 MAPK and p53 signaling pathway in GC. Ectopic expression (E1) represents gain-of-function (GoF) while knockdown (KO) represents loss-of-function (LoF) of the respective network element. The leftmost column shows the level of DNA damage with other molecules separated by a pink line. In contrast, the rightmost columns show the model outputs: proliferation, cell cycle arrest, and apoptosis. Each line represents a fixed point or steady state corresponding to the input. Light purple cells indicate a zero value (inactivation), while dark purple cells indicate activation (value 1). The first two fixed points are related to loss-of-function (LoF) of p53, that is, all node knockdown (KO) of p53. As you can see, only the ATM/p38 MAPK pathway is activated and triggers cell cycle arrest and apoptosis in response to DNA damage. The next two fixed points are described as loss-of-function (LoF) of p38 MAPK, that is, induction of proliferation. Apparently, the first fixed point knockdown (KO) of p38 MAPK represents a proliferation state due to the inactivation of DNA damage. Even in the presence of DNA damage, knockdown (KO) of p38 MAPK accelerated proliferation due to activation of cell cycle promoters such as E2F1, Myc, and CDK-cyclin complex. As you can see, loss-of-function (LoF) of p38 MAPK in response to DNA damage, lncRNA-GAS5 expression is accelerated but fails to trigger cell cycle arrest and/or apoptosis except in the proliferation state. After that, the other two fixed points represent the gain-of-function (GoF) of p38 MAPK, which means cell cycle arrest and apoptosis as well as activation of the p53 pathway. The ensuing fixed points are associated with gain-of-function (GoF) of p38 MAPK as well as loss-of-function of p53. Clearly, activation of the expression of the lncRNA-GAS5, miR-34c, p21, and RB1 triggers arrest and apoptosis (independently of p53). The other two following fixed points indicate the knockdown (KO) of ATM. Equally, this was observed in the loss-of-function (LoF) of p38MAPK. This means that at a certain point in the absence of DNA damage and another in response to DNA damage triggered proliferation; similarly, the lncRNA GAS was activated but refused to induce cell cycle arrest and apoptosis-like phenotypes. The other two fixed points of ATM represent the gain-of-function (GOF) of ATM, and then, more two fixed points (at last) represent the overexpression (E1) of ATM with p53 KO (knockdown), i.e., expression of the lncRNA-GAS5, miR-34c, p21, and RB1, induces cell cycle arrest and apoptosis (independently of p53). Yellow arrows represent upregulation, while the red arrows facing down represent downregulation, respectively.

Indeed, Liu et al.^7^ confirmed that G1/S arrest and apoptosis are independent of p53 but dependent on the p38 MAPK pathway in GC cell lines. Furthermore, Subhash et al.^8^ provide further evidence that ATM mediates p53-independent regulation of apoptosis and cell-cycle arrest at the G1/S checkpoint in GC cells. Interestingly, various studies have recently shown that lncRNA-GAS5 is associated with the ATM/p38 MAPK pathway^31–34^. To address whether this ATM/p38 MAPK signaling pathway, independent of p53 and can regulate cell cycle arrest and apoptosis at the G1/S checkpoint. We performed node perturbation to examine the agreement between the phenotypes (fixed points of the model) and in-vivo/in-vitro experiments. The results are presented in Fig. 3. As expected, we found that loss-of-function (LoF) of p38 MAPK or ATM, i.e., knockdown of p38 MAPK or ATM, induces GC proliferation. Whereas gain-of-function (GoF) of p38 MAPK and ATM triggers cell cycle arrest and apoptosis in GC cell lines. We conducted two separate perturbations of each node. In more detail, first, we performed the single node perturbation, i.e., we overexpressed (E1) of p38 MAPK, and second, double node perturbation, which means we performed loss-of-function (LoF) of p53 together with gain-of-function (GoF) of p38 MAPK. We repeated the same technique with ATMs. First, we overexpressed (E1) ATM alone, and next, we overexpressed (E1) ATM along with knockdown (KO) of p53. In this way, we can determine that ATM/p38 MAPK may regulate cell fate decisions such as cell cycle arrest and apoptotic cell death beyond the functionality of p53 in GC cell lines. Interestingly (see Fig. 3). We observed that p38 MAPK and ATM knockdown (KO) cells, lncRNA-GAS5, were stimulated in the presence of DNA damage; however, lncRNA-GAS5 failed to regulate cycle arrest and/or apoptosis. These results provide two important conclusions. First, the ATM/p38 signaling pathway, rather than p53, is required for modulating tumor growth and proliferation in GCs. Second, the lncRNA-GAS5 regulates cell fates in an ATM/p38 signaling pathway-dependent manner. Our results are in excellent agreement with the in-vivo/in-vitro experiments^7,8,31,33,34^.

### Clinical trials proposed by the Boolean network

The concept of experimental design is driven by Guo et al.^35^. Who found that lncRNA-GAS5 blocks the G1/S checkpoint by modulating CDK6. Indeed, forced expression of lncRNA-GAS5 enhanced p21 activation, which directly inhibits CDK4/6-CyclinD and CDK2-CyclinE at the G1/S checkpoint. However, so far, there is no study/evidence yet that can uncover the functional concrete relationship between lncRNA-GAS5 and miR-34c. Therefore, we propose a new clinical framework defined by the network that a lncRNA-GAS5 functioning as an inhibitor of E2F1 may inhibit cancer progression through the establishment of miR-34c and can induce cell cycle arrest and apoptosis at the G1/S checkpoint.

A conceivable outcome of this experiment would be the suppression of tumor growth and proliferation in GC. To test this development, we insist on these potential perturbations scenarios predicted by the network.

- lncRNA-GAS5 (E1) → E2F1 (KO) → miR-34c ↑
- lncRNA-GAS5 (E1) → E2F1 (KO) → RB1 ↑
- lncRNA-GAS5 (E1) → E2F1 (KO) → p21 ↑

## Discussion

It is widely known that E2F1 is involved in both cancer cell growth and apoptosis^36,37^. Petrocca et al.^38^ went into further depth, showing that transfection of E2F1 overexpression and suppressing expression in GC cells might promote cell proliferation and prevent apoptosis. Similar to this, Guo et al.^39^ recently discovered that miR-537/E2F1 has a positive (double negative) feedback loop which inhibits GC development and proliferation by activating cell cycle arrest and apoptosis. Furthermore, Zhang and colleagues^40^ discovered that overexpression of E2F1 and p53 cause cancer cells to undergo apoptotic cell death in response to DNA damage. We have also revealed the dual functionality of E2F1 in both proliferation and apoptosis in glioblastoma multiform (GBM)^41^. High expression of E2F1 has been linked to poor outcomes in GC^15^. As no genetic aberrations have been found so far that could describe the upregulation of E2F1 in GC^42^. The lncRNA-GAS5 is a key regulator of E2F1 expression and is downregulated in GC^15^. Similarly, miR-34c is downregulated in GC^3^. Interestingly, miR-34c was inhibited by E2F1 in GC^3^. It was unknown whether the lncRNA-GAS5/E2F1 axis plays an important role in the regulation of miR-34c in GC. Here, we constructed a Boolean network to identify lncRNA-GAS5 interactions in GC (see Fig. 1). The model suggested the lncRNA-GAS5 as a key regulator of miR-34c through its ability to repress E2F1 expression. Consistent with this concept, high expression of the lncRNA-GAS5/miR-34c in GC was correlated with suppression of GC progression and proliferation through induction of cell cycle arrest and/or apoptotic in GC^3,15^.

Our results suggest the mechanism by which increased expression of lncRNA-GAS5 may enhance miR-34c expression in GC. lncRNA-GAS5 targets E2F1 expression, which is the main inhibitor of miR-34c activity in GC. Knockdown of E2F1 was followed by increased expression of the tumor suppressor miR-34c, i.e., inhibition of tumor growth and proliferation at the G1/S checkpoint. The following assumption is confirmed by the observation of lncRNA-GAS5 expression at three fixed points (attractors). The results are shown in Fig. 2. In the first fixed point, lncRNA-GAS5/miR-34c was downregulated, whereas E2F1, CDK4/6-CyclinD, CDK2-CyclinE, Cdc25A were highly expressed. In response to DNA damage, two fixed points were found to be associated with lncRNA-GAS5, conferring constitutive activation of miR-34c in both fixed points. On the other hand, E2F1 was downregulated in both fixed points, which was expected.

Indeed, our Boolean network captured a specific molecular mechanism related to the lncRNA-GAS5 that had never been reported before. Nevertheless, all these marked results are established on the discrete core of the model elements. Therefore, the prediction of time-dependent features as the evolution of precise expression levels over time is one of the limitations of our model.

Plenty of studies are available that highlight the essential role of lncRNAs sponging miRNAs in controlling mRNAs. For example, numerous studies found that the lncRNA-GAS5 sponged miR-21^43^, miR-222^31^, and miR-106a-5p^44^ in several cancers, including GC. On the other hand, the lncRNA-GAS5 is also known to directly (positively or negatively) regulate mRNAs expressions such as E2F1^15^, p53^45^, and mTOR^46^. However, there is no evidence yet that lncRNAs can positively regulate miRNAs expression by sponging/decoys of other molecules (e.g., other lncRNAs, miRNAs, mRNA). Our results highlighted a very convincing relationship between the lncRNA-GAS5 and miR-34c via E2F1. Unraveling this novel molecular mechanism associating lncRNA-GAS5/miR-34c for the first time confirms that lncRNAs can positively modify miRNAs and that collectively they can effectively inhibit the tumor growth and proliferation in GC.

**Figure 4:**
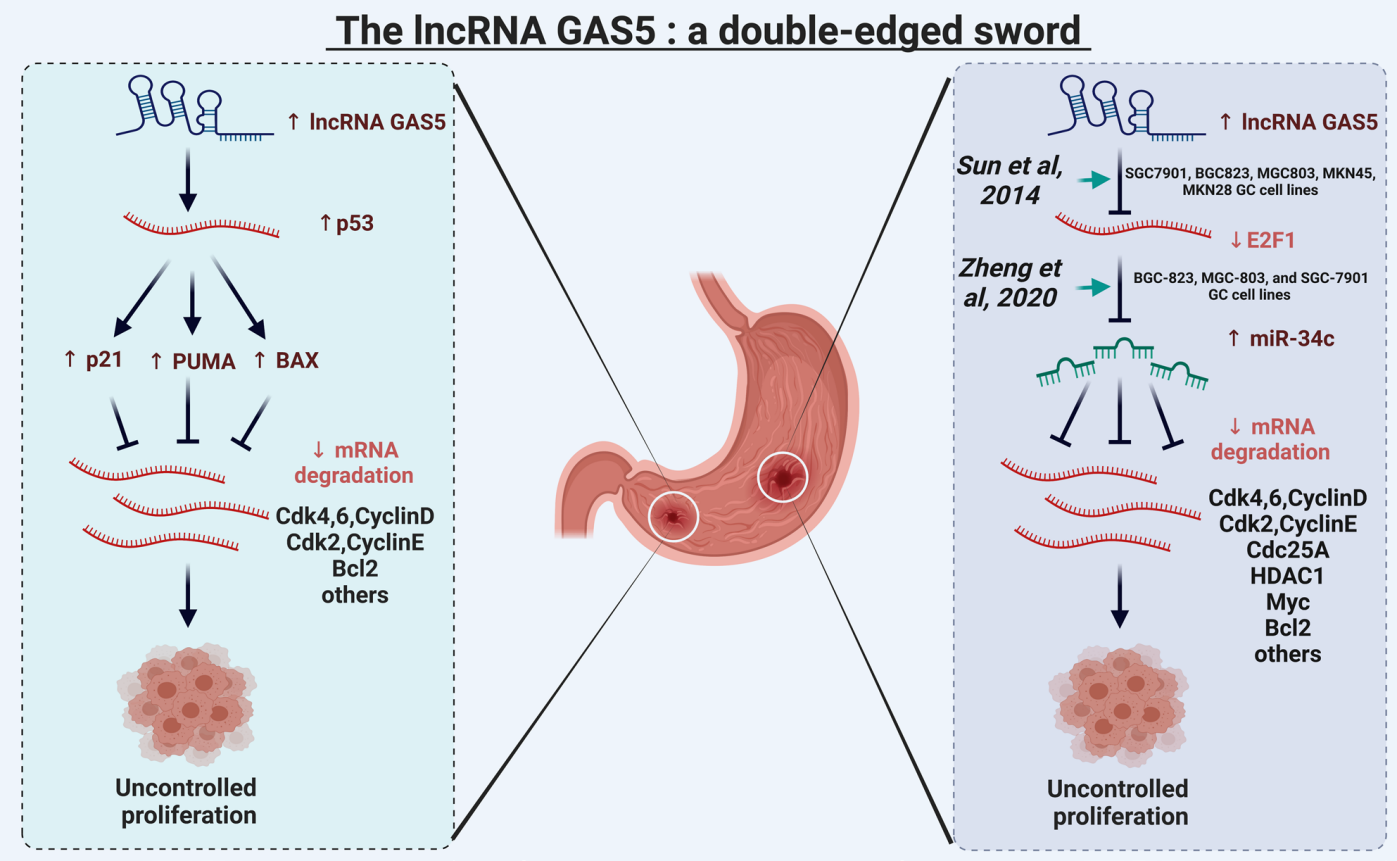
lncRNA-GAS5: a double-edged sword in tumor growth and proliferation in GC. On the left (highlighted in the green box), p53 was directly activated by lncRNA-GAS5 in GC^45^. Once p53 expression is activated, it may induce p21^68^, PUMA/BAX^68^. Although, the expression of p21 does not depend on the p53 functionality in GC^7,8^. Despite this fact, activated p21 and PUMA/BAX, which directly inhibit the expression of the CDK4/6-CyclinD and CDK2-CyclinE, Cdc25A, Bcl2, and others and induce cell cycle arrest and apoptosis at the G1/S checkpoint. On the right (highlighted in the purple box), the novel tumor suppressor mechanism of lncRNA-GAS5 is captured by the Boolean network. Overexpression of lncRNA-GAS5 directly inhibits E2F1 expression^15^, which triggers miR-34c expression^3^. Then, overexpression of miR-34c directly targets the CDK4/6-CyclinD and CDK2-CyclinE, Cdc25A, HDAC1, Myc, Sirt1, Mdm2, and Bcl2, see the review by Xiong et al.^67^. Targeting these molecules activates cycle arrest and apoptotic cell death at the G1/S checkpoint in GC. Thus, lncRNA-GAS5/miR-34c axis inhibits tumor growth and uncontrolled proliferation in GC. Consequently, p53-dependent or independently of p53, lncRNA-GAS5 can prevent tumor progression and cell proliferation in GC.

Additionally, our results revealed that the ATM/p38 MAPK pathways are essential for regulating cell cycle arrest and apoptosis at the G1/S checkpoint in GC cell lines^7,8^. It is well known that p53 is the main regulator of cell fate in response to DNA damage and plays an important role in controlling tumor growth^47^. However, in GC cell lines, p53 mutations have been found in up to 70%^48^, and there is much evidence confirming that the ATM/p38 MAPK pathway compensates for p53 deficiency in GC cells^7,8,49^. So, we tested it, and the results are shown in Fig. 3. We found that cell fate decisions such as cell cycle arrest and/or apoptosis are independent of p53 status in GC cells as suggested by these in-vivo/in-vitro studies^7,8,49^. Although WT p53 is defined in some GC cell lines, it will induce cell cycle arrest and/or apoptosis, as both ATM/p38 MAPK are upstream regulators of p53^50,51^. Whereas p53-deficient GC cell lines, ATM/ p38MAPK pathway eliminates the tumor growth and proliferation by the activation of cell cycle arrest and apoptosis in GC cell lines. Thus, lncRNA-GAS5 acts as a double-edged sword in GC progression through the activation of miR-34c expression as well as p21 and RB1. For more detail, see Fig. 4. Consequently, Our results provide a broad and relevant background in this new direction. miRNAs are already well-known players in cancer research^52^. The molecular mechanisms associated with lncRNAs are not yet fully understood, and there is still much to be revealed. The results presented in this work will provide a new direction to many researchers with diverse expertise in fighting cancer (development of new technologies and new experiments) based on accumulated knowledge.

In summary, we developed a Boolean model of lncRNA-GAS5 at the G1/S checkpoint based on the literature and public databases in GC. Our Boolean model simulations uncover very complex scenarios between lncRNA-mRNA-miRNA, i.e., lncRNA-GAS5, E2F1, and miR-34c in response to DNA damage. Our results suggested that lncRNA-GAS5 is an important regulator of miR-34c activity in GC. Thus, our approach may contribute to proposing alternative therapeutic strategies for cancer through drugs targeting this lncRNA-mRNA-miRNA axis that might significantly reduce the tumor growth and proliferation in GC cancer cells.

## Methods

### Development of the gene regulatory network and collection of the public databases & tools

Building a gene regulatory network of lncRNA-GAS5 activity GC cells. We have only used PubMed studies^53^ and databases such as BioGrid^54^. The focus was to identify genes or proteins that were targeted by the lncRNA-GAS5 (Fig. 1), such as E2F1 and cyclin D1. Furthermore, we identify genes or proteins that are directly targeted through the miR-34c. For that, we have used public databases like; miRTargetLink^55^ and TargetScan^56^, respectively.

GINsim 3.0.0b was used for the construction and simulation of the Boolean network and visualization of the results^57^, which is a Java-based software and is freely available to researchers (http://www.ginsim.org/downloads). GINsim algorithms recognize all the attractors for the wild-type case as well as for various mutant situations^57^.

The model file is available in the “Code availability” section.

### Translation of PubMed literature into boolean rules and simulations of the boolean network

The Boolean method is based on the characterization of a regulatory graph, where an individual node defines a molecule, and each directed edge (or arc) signifies an activation or inhibition among two nodes. Nodes are Boolean variables that only consider 0/OFF and 1/ON values. Based on the description of the biochemical information, each node in the network is assigned a logical rule, which determines its activation level concerning the position of its regulators^58^.

Our Boolean network model of lncRNA-GAS5 was produced by translating the biological interactions of the DNA damage-induced G1/S checkpoint, which is an ATM/p53-dependent, described in the gene regulatory network (Fig. 1) into Boolean rules. For more details, see Supplementary Table S1 (PubMed links included). Classical Boolean operators were used to write these rules “AND,” “OR,” and “NOT”. Attractors are the main outcome of simulations using a Boolean network^58^. The dynamical performance of a Boolean model can be interpreted by a state transition graph (STG). In this graph, each node describes the state of the network variables, and the arcs signify transitions between these states^58^. The STG serves all possible trajectories that one initial state can drive to a final state. Terminal nodes that have no outgoing edges are called stable states (or fixed points), while a set of transitions trapped among a fixed group of states in the STG defines a cyclic state^58^. For the updates of states, synchronous updates were considered, which has the potential to describe deterministic behavior observed in molecular networks^16,57^. In addition, in silico Gain-of-Function (GoF) or Loss-of-function (LoF) perturbations, we force node values to be ON or OFF, respectively, to examine the effect of particular nodes on network dynamics and the resulting phenotype^57,58^.

### Expected performance of the lncRNA-GAS5 Model

Simulations using the lncRNA-GAS5 Boolean network should describe the biology of a cancer cell (with or without DNA damage in GC). Indeed, cancer cells can be simplified as either remains a proliferative state due to downregulation of lncRNA-GAS5/miR-34c^3,15^ i.e., upregulation of E2F1^15^ (in the absence of DNA damage). Whereas, in the case when DNA damage is present in the cell, upregulation of lncRNA-GAS5/miR-34c is required that can repress the proliferation by the induction of cell fate decisions such as cell cycle arrest and/or apoptosis at the G1/S checkpoint^3,14,15^. Consequently, at least three fixed points/stable states (attractors) are expected.

### lncRNA-GAS5-mediate molecular mechanism of the G1/S checkpoint in GC

It is well-known that cell fate decisions such as cell cycle arrest or apoptosis are induced by DNA damage response at both G1/S and G2/M checkpoints^22,59–61^. Interestingly, lncRNA-GAS5 can be induced by DNA damage as suggested by Liu et al.^14^ and Zhou et al.^62^. In addition, in the event of the G1/S checkpoint, about 20 molecules are involved in its main regulatory network^63^. DNA double-strand breaks (DSBs) can be affected by the radiomimetic chemicals or reactive oxygen species (ROS) or radiation, which triggers autophosphorylation of ATM at serine 1981 by initiating its kinase activity^63,64^. Downstream phosphorylation at the ATM pathway leads to the activation of p53 in response to DNA damage^64^. In GC cells, lncRNA-GAS5 is required for the induction of the G1/S checkpoint, which is our focus here; in fact, GC cells knocked down for lncRNA-GAS5 cannot arrest at the G1/S phase^15^.

In DNA damage response, In this model, based on its different phosphorylation events, p53 is denoted by different nodes^65^. The p53 node is linked with its interaction with Mdm2, which is required to initiate p53-A and p53-K^65^. p53-A represents p53 phosphorylated at Ser-15 and Ser-20 that activated p21, while p53-K represents p53 further phosphorylation at Ser-46, which leads to the activation of the apoptotic pathway via BAX^65^. P53-A and p53-K are attached by a positive circuit^65^, and the conversion between p53-A and p53-K is ruled by the protein phosphatase 1D (Wip1) and the tumor protein p53 inducible nuclear protein 1 (p53-INP1)^65^. In the network, apoptosis is triggered by caspase, which is regulated by Bcl2, Bax, and p21. In more detail, activation of Bax and inactivation of Bcl2 and p21 are associated with caspase activation, which causes apoptotic cell death.

In addition, p53 is a well-known activator of the miR-34 family (miR-34a, miR-34b, and miR-34c) in the DNA damage response^66^. However, the expression of miR-34c modulates by ATM and p38 MAPK pathway, which is independent of de-novo p53^4,5^. Therefore, in our network, miR-34c is directly activated by the ATM/p38 MAPK pathway^4,5^ or indirectly by the lncRNA GAS5/E2F1 pathway^3,15^.

miR-34c directly targets E3 ubiquitin-protein ligase Mdm2 (Mdm2), Myc proto-oncogene protein (Myc), Cyclin-dependent kinase 4/6 (cdk4/6-Cyclin-D), and the Cyclin-dependent kinase 2-G1/S-specific cyclin-E1 (cdk2/Cyclin-E) complex, Cell division cycle 25A (Cdc25A), NAD-dependent protein deacetylase sirtuin-1 (Sirt1), Histone deacetylase 1 (HDAC1), and Apoptosis regulator Bcl-2 (Bcl2), for more details about miR-34c targets see review by Xiong et al.^67^. Worth noting is that in the lncRNA-GAS5 Boolean network, we selected only experimentally verified targets of miR-34c. Forced expression of miR-34c induces cycle arrest by targeting cdk4/6-Cyclin-D, cdk2/Cyclin-E complex, HDAC1, Myc, and Cdc25A^3,66^, whereas it triggers apoptotic phenotype by knockdown Bcl2^3,66^.

## Supplementary Information

Supplementary Table S1.

## Data Availability

All data needed to evaluate the conclusions in the paper are present in the paper and/or the Supplementary Materials.

## Code availability

The model code is available in the Github repository: https://github.com/pritampanda15/lncRNA-GAS5.
